# Growth and development of *Gnathostoma spinigerum *(Nematoda: Gnathostomatidae) larvae in *Mesocyclops aspericornis *(Cyclopoida: Cyclopidae)

**DOI:** 10.1186/1756-3305-4-93

**Published:** 2011-05-27

**Authors:** Penchom Janwan, Pewpan M Intapan, Oranuch Sanpool, Luxkhana Sadaow, Tongjit Thanchomnang, Wanchai Maleewong

**Affiliations:** 1 Department of Parasitology, Faculty of Medicine, Khon Kaen University, Khon Kaen 40002, Thailand; 2 Research and Diagnostic Center for Emerging Infectious Diseases, Faculty of Medicine, Khon Kaen University, Khon Kaen 40002, Thailand; 3 Faculty of Medicine, Mahasarakham University, Mahasarakham 44000, Thailand

## Abstract

**Background:**

*Gnathostoma spinigerum *larva is pathogenic, causing gnathostomiasis in humans and certain animals, and is prevalent mainly in Asia. Growth and development of *Gnathostoma spinigerum *larvae in the cyclopoid copepod *Mesocyclops aspericornis*, the first intermediate host, were examined.

**Results:**

When newly hatched, ensheathed second-stage larvae (L2) were ingested by *M. aspericornis*, they immediately appeared exsheathed in the stomach of *M. aspericornis*. They then penetrated the stomach wall and entered the body cavity, where they immediately metamorphosed to a stunted form with the body length/width ratio equal to the early third-stage larvae (EL3) up to 2 h after being ingested. During metamorphosis, the anterior spine-like structure of L2 transformed into unequal transparent lips. The larvae moulted into EL3 in the body cavity of the copepod at around day 5-7 post-infection. Minute cuticular striations were seen on the whole body, with prominent single-pointed spines on the anterior part of the body. The head bulb had four rows of hooklets and two lateral trilobed lips. The size of EL3 in copepods continuously increased towards day 12 and showed a negative correlation to their density per copepod (R = -0.881, *P *< 0.05 for body length, and R = -0.906, *P *< 0.05 for body width).

**Conclusions:**

The results revealed for the first time that *M. aspericornis*, one of the most abundant freshwater copepods in Thailand, is a suitable first intermediate host for *G. spinigerum*. High susceptibility of *M. aspericornis *suggests its importance for the maintenance of the life cycle of *G. spinigerum *in Thailand.

## Background

*Gnathostoma spinigerum *is a nematode parasite mostly found in the stomach wall of canine and feline definitive hosts. *Gnathostoma spinigerum *larvae are also pathogenic, causing gnathostomiasis in humans and certain animals, and is prevalent mainly in Asia [1,2]. The life cycle of *G. spinigerum *has been previously reported [1,2]. When eggs passed in feces reach freshwater, the second-stage (L2) larvae develop in ovo within one week, hatch from eggs, and begin swimming freely. Once L2 are eaten by cyclopoid copepods, the larvae cast their sheath and develop into complete L2 and early third-stage larvae (EL3). When the infected copepods are ingested by various intermediate/paratenic hosts - e.g. fishes, amphibians, reptiles, etc, the larvae develop further to become advanced third-stage larvae (AL3), which infect the definitive hosts. When AL3 are ingested by the definitive hosts, they migrate further into the host tissue and finally develop into the adult stage in the stomach wall to complete their life cycle. Cyclopoid copepods, thus, play a crucial role in maintain the life cycle of *G. spinigerum*. In Thailand, 4 species of cyclopoid copepods, *Mesocyclops leuckarti*, *Eucyclops agilis*, *Cyclops varicans*, and *Thermocyclops *sp. were proven experimentally as the first intermediate host for *G. spinigerum *[3]. Here, we report for the first time that *G. spinigerum *can develop into EL3 in *M. aspericornis*, the pantropical freshwater cyclopoid copepod widely spread in Asia, America and Africa [4-6], and is abundant in Thailand [7]. High susceptibility of *Mesoycyclops *suggests its importance for the maintenance of the life cycle of *G. spinigerum *in Thailand.

## Methods

### Parasite

Eggs of *G. spinigerum *were collected from the stools of experimentally infected dogs by a brine floatation method [8]. Each animal was housed in a kennel (6.5 × 6.5 m^2^/dog) with a concrete floor and wooden stand for rest; it was cleaned daily with a high-pressure cleaner. Dogs were fed with commercially prepared dry dog food and had access to tap water *ad libitum*. The study was carried out at the experimental unit of the Faculty of Medicine, Khon Kaen University (Khon Kaen, Thailand). After extensive washing, eggs were suspended in freshwater and incubated at 27 ± 2°C (mean ± SD). Development of embryonated eggs was regularly monitored. After hatching, L2 were collected for experimental infection to copepods. The study protocol was approved by the Animal Ethics Committee of Khon Kaen University, based on the Ethics of Animal Experimentation of the National Research Council of Thailand (Reference no.0514.1.12.2/1).

### Mass rearing of cyclopoid copepods

Copepods were collected from their natural breeding site at Muang district, Khon Kaen province in northeastern Thailand and were identified as *M. aspericonis *by using the descriptions and keys of Van de Velde [4]. Several isofemale lines were established from gravid females and maintained in the Department of Parasitology, Faculty of Medicine, Khon Kaen University. Gravid female *M. aspericornis *from different isofemale lines were mass-reared in dechlorinated water in 1 L beakers (15 isofemale lines/beaker) at 27 ± 2°C. *Paramecium *spp., cultured in boiled rice straw water extract, and commercial powdered fish food were used to nourish the copepods.

### Experimental infection of copepods with *G. spinigerum *larvae

*M. aspericornis*, 14-day-old, were placed in clean 600-ml beakers containing 250 ml dechlorinated water (80 copepods/beaker). They were divided into three duplicate groups, and each duplicate group was fed with 400, 700 and 1,200 *G. spinigerum *L2. The experiment was terminated 12 d after infection.

### Development of *G. spinigerum *larvae in copepods

Infected copepods were sampled and dissected under a stereomicroscope. Body length and width of *G. spinigerum *L2 and EL3 were measured daily up to day12 post-infection (PI). All recovered larvae were photographed under a microscope (Olympus BX51, Tokyo, Japan). Worm length and width were determined using a DP2-BSW XV image processing program (Olympus) and calibrated with the stage micrometer. Body length was measured from the head lip to the end of the tail, while body width was measured at the esophageal-intestinal junction. To explore the density effect of EL3 on their infectivity, the large EL3 were recovered from copepods harboring only 1 larva, whereas the small EL3 were recovered from copepods harboring 13 larvae on day 12 PI. They were separately fed to mice orally (15 EL3/mouse; 6 mice/group). All mice were killed by ether inhalation on day 30 PI and dissected. All organs were examined under a dissecting microscope (14-45×) for AL3 by compression method, and the mean number and size of AL3 from each group were determined.

### Statistical analysis

Results were analyzed statistically by Student's *t*-test or Mann-Whitney rank sum test, as appropriate; a *P *value less than 0.05 was considered significant. Correlations were analyzed using Spearman's rank correlation test.

## Results

### Development of *G. spinigerum *larvae in copepods

**Figure 1:**
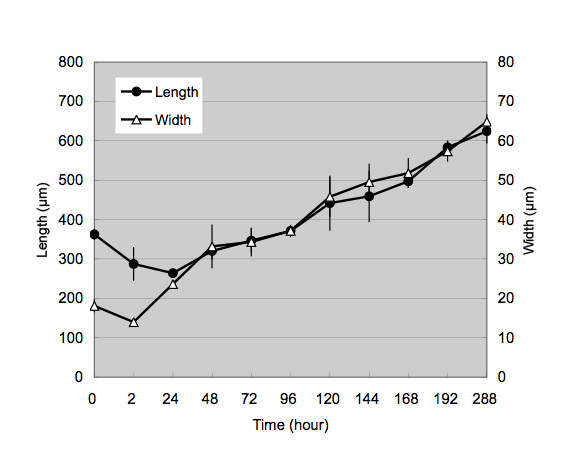
**Chronological changes of body sizes**. The chronological changes of *Gnathostoma spinigerum *body sizes in *Mesocyclops aspericornis*.

Growth and development of newly hatched *G. spinigerum *larvae in *M. aspericornis *was monitored immediately after ingestion up to day 12 PI. The chronological changes of the body sizes are summarized in Figure 1. The newly hatched L2 was slender filariform about 18 × 360 μm (width × length) and were enclosed in a delicate, smooth, transparent sheath. The round anterior head was armed with a minute, solid spine-like structure.

Immediately after being ingested by copepods, the larvae exsheathed to become the complete L2 with the reduced size. Two small transparent unequal lips developed at the head of L2 at around 1 h PI. L2 in the stomach of copepods further reduce their size measuring about 14 × 280 μm at 2 h PI. Then they penetrated across the stomach wall to lodge in the body cavity of copepods by 24 h PI where they started to increase in width but further reduce in length slightly, measuring about 24 × 264 μm. At this stage, granulated cells were observed in the gut lumen of L2. Hereafter, the body width:length ratio remained about 1:10, regardless of L2 or EL3 stages.

**Figure 2:**
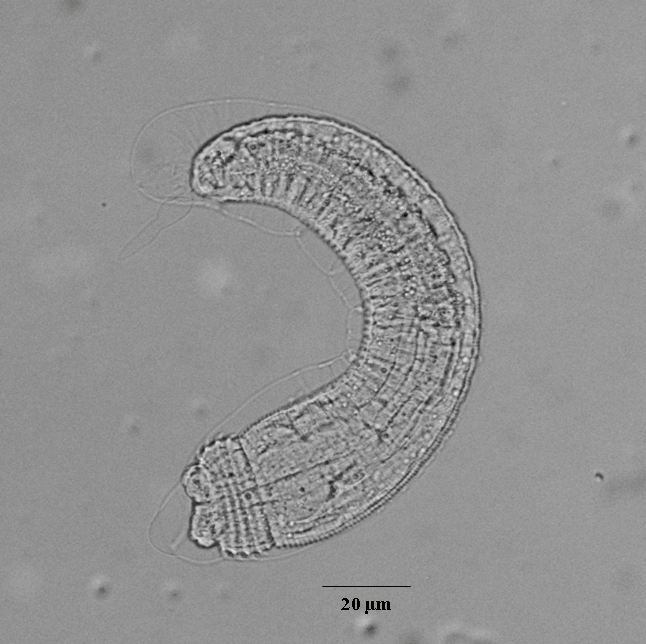
**Five-day-old *G. spinigerum *larva**. The molted cuticle extending slightly beyond the head and tail ends.

On day 2 PI, the overall size of larvae increased (33 × 320 μm), and early development of cervical sacs was seen. On day 3, the body size increased further (34 × 346 μm), and the esophagus and intestine became visible. On days 4-5 PI, the larvae showed continuous growth and the cells of cervical sacs, esophagus and intestine were distinctly seen. On day 5 PI, transverse striations of the body cuticle and head bulb were formed. Some larvae showed a molting cuticle (sheath) extending slightly beyond the head and tail ends (Figure 2).

On day 6, the larvae further increased in size (50 × 460 μm), and the majority of larvae exsheathed to become early third-stage larvae (EL3). Minute cuticular striations were observed over whole body with prominent single-pointed spines on the anterior part of the body. Two lateral trilobed lips were symmetrically formed. The head bulb had four rows of hooklets. Cervical glands and four cervical sacs were distinctly formed; the esophagus, intestine and anus were also well-defined. The posterior end of the larval worm was round in shape. The undifferentiated genital primordium was located near the esophageal-intestinal junction. All these features are characteristic of EL3.

On day 7 PI, all larvae examined were exsheathed. EL3 showed sluggish movement within the body cavity of copepods (See additional File 1). On days 8-12, the larvae did not show any significant morphological transformation but consistently increased their sizes. On day 12 PI, they measured 65 × 625 μm.

### Density effect on the growth/development of larvae

**Figure 3:**
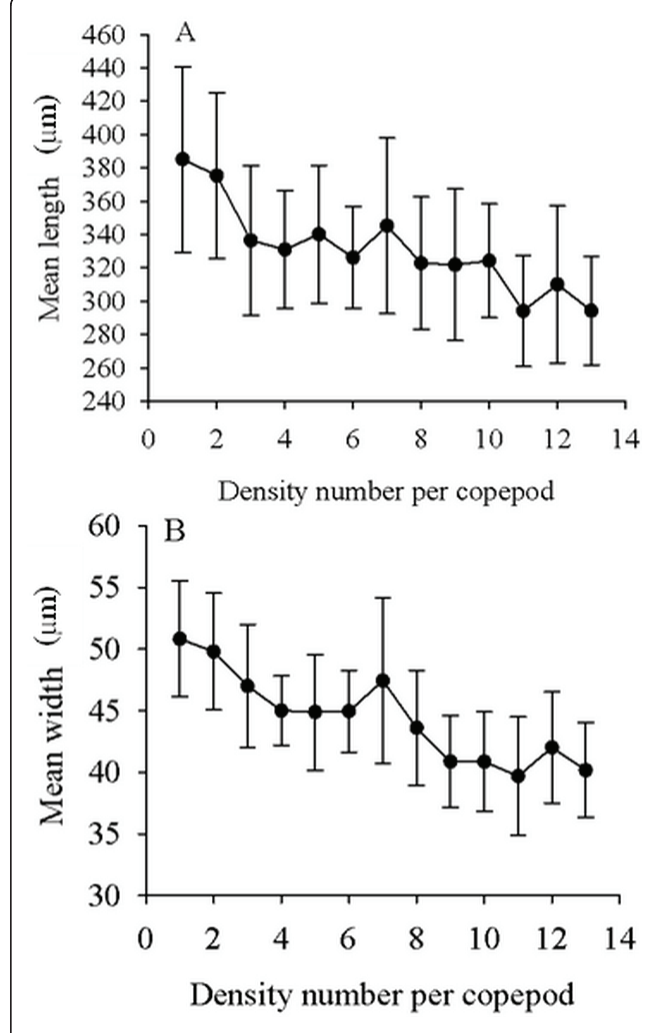
**Relationship of worm size and density per copepod**. The relationship of worm size and density per copepod: **A**, length; **B**, width.

All infected copepods were dissected on day 12 PI. The intensity of infection per copepod ranged from 1 to 13. Mean (±SD) body length and width of EL3 in relation to the number of larvae per copepod are shown in Figure 3. A negative relationship (R = -0.881, *P *< 0.05 for body length, and R = -0.906, *P *< 0.05 for body width) was observed between the mean length and width of EL3 and the density of larvae in copepods.

To see whether the initial size of EL3 would affect the subsequent growth/development to the advanced third-stage larvae (AL3) in mammalian hosts, the small larvae collected from heavily infected copepods (13 EL3/copepod) and the large larvae collected from copepods having single EL3 were separately infected in mice (15 EL3/mouse for both group) and the AL3 were recovered on day 30 PI. The results were summarized in Table 1. For both groups of mice given small or large AL3, the mean number ± SD of worm recovery per mouse were 5.50 ± 1.04 and 5.67 ± 1.21, respectively (*P *> 0.05). Approximately 40% of EL3 developed into AL3 in both groups. The body length and width of AL3 descended from small and large EL3 from copepods were also not significantly different regardless of the size of the ancestral EL3 (Table 1).

**Table 1:** Effect of initial size of EL3 on the subsequent development to AL3 in mice

	Small EL3	Large EL3	*P *value
AL3			
Recovery (No./mouse)	5.67 ± 1.21	5.50 ± 1.04	>0.05
Length (μm)	3863.22 ± 365.39	3773.32 ± 458.64	>0.05
Width (μm)	330.40 ± 27.83	331.11 ± 25.90	>0.05

EL3: early 3^rd ^stage larvae, AL3: advanced 3^rd ^stage larvae

## Discussion

In this study, we extensively observed the growth and development of *G. spinigerum *larvae in a first intermediate host, the copepod *M. aspericornis*. Previously *M. leuckarti*, *E. agilis*, *E. serrulatus*, *C. varicans, C. strenuus, C. vicinus *and *Thermocyclops *sp. [1,3,9,10] have been proven as experimental first intermediate hosts for *G. spinigerum *larvae. Our results revealed for the first time that *M. aspericornis*, one of the most abundant freshwater copepods [4-6] - especially in Thailand [7] - is also a suitable first intermediate host for *G. spinigerum*. To our knowledge, *Gnathostoma *larvae have never been found in any copepod species in the wild. *M. aspericornis *should be included as a potential candidate of the natural first intermediate hosts for *G. spinigerum*.

*Mesocyclops aspercornis *is a cyclopoid copepod abundant in Afro-Asian continents [4-6]. Moreover, this species has been artificially propagated to control mosquito larvae in natural freshwater reservoirs in the dengue vector control program in various countries [11-13] including Thailand [7]. Such an artificial expansion of *Mesocyclops *might cause contamination of natural water reservoirs and would cause acceleration of the *G. spinigerum *life cycle or create new endemic areas of gnathostomiasis. From a public health point of view, the prevalence of *G. spinigerum *larvae in fish and the human habits of consuming raw or undercooked fish should be examined before introduction of the mosquito control programs by *Mesocyclops *to avoid possible outbreaks of gnathostomiasis.

In the present study, the growth/development of *G. spinigerum *larvae in *M. aspericornis *were basically identical to that reported by Miyazaki [1,9], and similar to that reported for other species, i.e. *G. binucleatum *[14], *G. turgidum *[15]*, G. hispidum *[3,16,17], *G. nipponicum *[18], and *G. procyonis *[19]. While the first molt from first-stage larvae to L2 occurred in eggs, L2 showed considerable reduction of body size and morphological changes during the first 2 h in copepods. The second molt from L2 to L3 required a longer incubation period, occurring at around days 5-7 PI and the body size continuously increased even during molting.

On day 12 PI, a negative relationship was observed between the density and the size of larvae in copepods. Density-dependent growth/development can occur at each stage of the parasite life cycle [20,21] and various model systems have been reported to investigate this phenomenon; *Trichostrongylus retortaeformis *infection in rabbits [22]; *Maritrema novaezealandensis *in amphipod hosts [23]; *Pomphorhynchus laevis *in their amphipod intermediate hosts *Gammarus pulex *[24]. Shostak et al. [25] revealed a density-dependent reduction of the body size of the tapeworm *Triaenophorus crassus *procercoid in the copepod *Cyclops bicuspidatus thomasi*, and explained this phenomenon by the limitation of the host size. However, in the present study, the sizes of AL3 descended from small and large EL3 from copepods were not significantly different regardless of the size of the ancestral EL3. This result should be further investigated. In addition, *G. spinigerum *larvae in the first intermediate host copepod can serve as a possible model that provides a better understanding of the ecological relationship between parasites and hosts.

## Conclusions

Our results revealed for the first time that *M. aspericornis*, one of the most abundant freshwater copepods in Thailand, is a suitable first intermediate host for *G. spinigerum*. Detailed growth and developmental process of *G. spinigerum *larvae in *M. aspericrnis *are described. A negative relationship was observed between the mean length and width of EL3 and the density of larvae in copepods.

## Competing interests

The authors declare that they have no competing interests.

## Authors' contributions

PJ carried out the experimental studies, participated in conception and design, and acquisition of data. OS and LS participated in conception and design and acquisition of data. TT participated in the design of the study and performed the statistical analysis. PMI carried out in revising it critically for important intellectual content and conceived of the study. WM participated in its design and coordination and helped to draft the manuscript. All authors read and approved the final manuscript.

## Supplementary Material

Additional file 1 **Video showing movement of *G. spinigerum *larvae in a copepod**. Click here for file
